# Substance P and Neurokinin 1 Receptor in Chronic Inflammation and Cancer of the Head and Neck: A Review of the Literature

**DOI:** 10.3390/ijerph19010375

**Published:** 2021-12-30

**Authors:** Francisco Esteban, Pablo Ramos-García, Miguel Muñoz, Miguel Ángel González-Moles

**Affiliations:** 1 Department of Otolaryngology, Hospital Universitario Virgen del Rocío, University of Sevilla, 41004 Sevilla, Spain; festebano@gmail.com; 2 School of Dentistry, University of Granada, 18010 Granada, Spain; magonzal@ugr.es; 3 Instituto de Investigación Biosanitaria ibs.Granada, 18012 Granada, Spain; 4 Research Laboratory on Neuropeptides (IBIS), Virgen del Rocío University Hospital, 41013 Sevilla, Spain; mmunoz@cica.es

**Keywords:** substance P, NK1R, head and neck cancer, squamous cell carcinoma, chronic inflammation

## Abstract

Head and neck cancer is a growing worldwide public health problem, accounting for approximately 1,500,000 new cases and 500,000 deaths annually. Substance P (SP) is a peptide of the tachykinin family, which has roles related to a large number of physiological mechanisms in humans. The implications of SP in carcinogenesis have recently been reported through the stimulation of the neurokinin 1 receptor (NK1R), or directly, through the effects derived from the constitutive activation of NK1R. Consequently, SP/NK1R seems to play relevant roles in cancer, upregulating cell proliferation, cell migration and chronic inflammation, among other oncogenic actions. Furthermore, there is growing evidence pointing to a central role for SP in tumour progression, singularly so in laryngeal and oral squamous cell carcinomas. The current narrative review of the literature focuses on the relationship between the SP/NK1R system and chronic inflammation and cancer in the head-and-neck region. We described a role for SP/NK1R in the transition from chronic inflammation of the head and neck mucosa, to preneoplastic and neoplastic transformation and progression.

## 1. Introduction

Head and neck cancer is a growing worldwide public health problem, with approximately 1,500,000 new cases and 500,000 deaths per year, mainly distributed across the oral cavity, pharynx, larynx and thyroid gland (GLOBOCAN, IARC, WHO) [1]. Head and neck cancer is a heterogeneous disease in clinical, pathological and molecular terms. Studies designed for diagnostic, prognostic and therapeutic purposes are identifying emerging molecular biomarkers acting as oncogenes and tumour suppressor genes with potential implications for the hallmarks of cancer (e.g., sustaining proliferative signalling or inflammation [2]). These emerging biomarkers could serve, in the future, as complements to clinical practice [3,4]. Recent evidence suggests that substance P (SP)/neurokinin 1 receptor (NK1R) alterations could play a key role in head and neck oncogenesis, particularly in laryngeal carcinomas and oral squamous cell carcinomas [5].

SP is a peptide of the tachykinin family, the potential roles of which are related to a large number of physiological mechanisms [6]. This peptide is present in both the central and peripheral nervous systems and is also widely distributed in diverse cell lineages (e.g., immune cells, lung, liver, etc.). SP, which binds NK1R, is also widely distributed and expressed in human tissues, regulating many biological and pathological roles, such as neuronal cell degeneration or death, the regulation of cardiovascular, arterial, respiratory, musculoskeletal and gastric systems, sensory perception, salivation and pain and inflammation [7]. SP also has an oncogenic function through the activation of NK1R, or directly, through effects derived from the constitutive activation of the NK1R receptor, such as inducing mitogenic pathways and sustaining proliferative signalling through the activation of the PI3K and MAPK oncogenic pathways [8,9,10,11,12], itself the canonical and most representative hallmark of cancer. Furthermore, both SP and NK1R seem to exert additional relevant roles linked to other canonical and emergent hallmarks of cancer, e.g., increased cell migration and invasion, resistance to cell death, cellular energetics deregulation, neoangiogenesis and chronic inflammation [9,13,14,15,16,17,18]. Consequently, SP/NK1R overexpression and oncogenic roles have been reported in several human neoplasms, such as brain tumours [9], leukemia [19], pancreatic [14], breast [16], endometrial [15] and head and neck cancers [5]. In this sense, our research group have reported promising findings in premalignant and malignant epithelia of the head and neck region [20,21,22,23]. Furthermore, the importance of NK1R upregulation in the head and neck also implies an opportunity to research and develop new targeted therapy strategies directed to NK1R. In this context, recent advances have shown a promising translational potential for Aprepitant, a NK1R-specific inhibitor, in a wide variety of cancers, including head and neck cancer [18].

Thus, based on this background, this narrative review of the literature focuses on the relationship between the SP/NK1R system and chronic inflammation and cancer in the head and neck region. We describe a role for SP/NK1R in the transition from chronic inflammation of the head and neck mucosa to preneoplastic and neoplastic transformation and progression.

## 2. SP/NK1R in Head and Neck Mucosal Inflammation and Cancer

It is accepted that, in 1863, Rudolf Virchow, noting leucocytes in malignant tumours, made a the first connection between cancer and inflammation [24]. Currently, several inflammatory conditions predisposed to cancer, as well as with other well-known associations, have been documented between chronic inflammation and malignant tumours, e.g., asbestos-induced inflammation and mesothelioma or bronchogenic carcinoma [25]; hepatitis B virus-related liver cirrhosis and hepatocellular carcinoma [26]; helicobacter pylori infection and gastric adenocarcinoma [27]; chronically inflamed mucosa in bowel disease and colorectal cancer [28]; HPV-related carcinogenesis and head and neck cancer [29]; tobacco smoking associated with both laryngeal chronic inflammation and laryngeal carcinoma [30]; and oral cancers’ possible development in the context of oral, potentially malignant disorders with a well-known inflammatory etiology [31], such as oral lichen planus [32]. In summary, growing evidence derived from experimental and clinical observations suggest a strong link between chronic inflammation and cancer [33]. Inflammation is considered a hallmark of cancer and contributes to the acquisition of multiple oncogenic capabilities by means of several molecular mechanisms [2], e.g., supplying molecules to the tumour microenvironment, such as growth factors, through tyrosine kinase receptors’ transactivation, mediating tissue repair in an inflammatory context (which increases mitogenesis and mutagenesis, and, consequently, sustaining proliferative signalling pathways [34,35,36]) or cytokines and chemokines, which may facilitate cancer growth, invasion and metastasis through several oncogenic mechanisms, such as the inhibition of DNA repair via reactive oxygen species, the inactivation of tumour suppressor genes or the stimulation of angiogenesis [24,37,38].

Inflammation is also relevant in head and neck cancer development [39]. Cancer in the head and neck region, when singular in the oral cavity, is often preceded by potentially malignant disorders, such as leukoplakia [40], erythroplakia [41], lichen planus [42], proliferative verrucous leukoplakia [43],or oral submucous fibrosis [44], the malignant transformation risk of which, currently evaluated according to the presence and severity of epithelial dysplasia [45], is variable depending on the clinical type [31]. The role of the inflammatory infiltrate has been proposed as a possible molecular bases for an epithelium prone to malignant transformation in oral lichen planus [46]. The actions of cyclooxygenase-2, produced by the inflammatory infiltration of cells that intervenes in the metabolism of arachidonic acid, could generate the carcinogenic metabolite malondialdehyde, consequently exerting DNA damage [46,47]. Resistance to apoptotic phenomena and an increase in the proliferation rate, as a consequence of stimuli from the inflammatory infiltrate itself, has also been reported in patients with oral lichen planus [46,48]. Nevertheless, these molecular events could also play oncogenic roles in other oral, potentially malignant disorders, such as proliferative verrucous leukoplakia, where the presence of a band-like lymphocytic chorionic infiltrate constitutes an unspecific histopathologic feature affecting up to 30% of patients [49]. Furthermore, increased levels of cytokines (e.g., interleukin-6 and tumour necrosis factor alpha) have also been reported in patients with oral lichen planus [50], oral submucous fibrosis [51] and oral leukoplakia [52]. In head and neck cancer, as previously mentioned, inflammation is a well-established hallmark, and numerous oncogenic mechanisms have been documented [39], e.g., the infiltration of inflammatory cells facilitating tumour development through the regulation of growth factors from mitogenic signalling pathways [53], the attenuation of the host immune response to tumour cells [54], or the determination of cytokines and chemokines playing key roles by promoting angiogenesis, proliferation and the immune cell response [55,56,57].

The SP/NK1R complex has been shown to play a key role in the microenvironment of inflammation and cancer [58]. SP/NK1R overexpression has also been documented in several of the previously mentioned inflammatory conditions with a higher risk of cancer predisposition, e.g., during inflammatory bowel disease—singularly, in ulcerative colitis—which has also been associated with an advanced severe clinical stage and a higher risk of colorectal carcinoma development [59,60,61]. Thus, it seems logical to propose that the SP/NK1R system, which is up-regulated in chronic inflammation processes, may play a role in the development of cancer [8,10,11,62,63,64,65].

**Figure 1:**
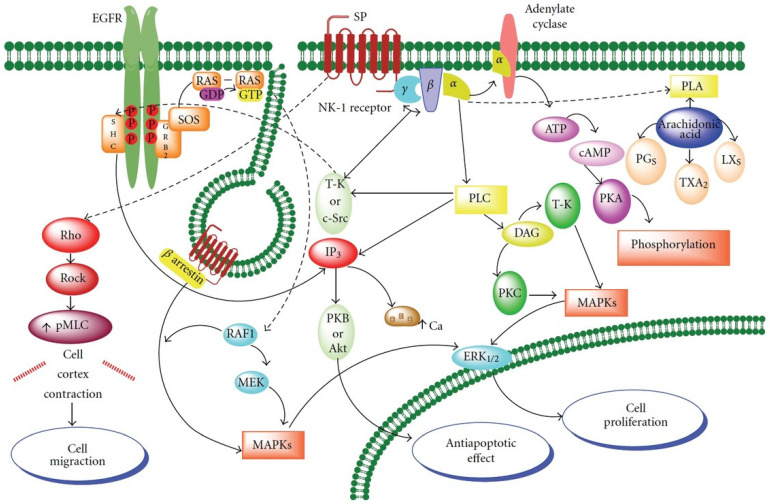
Signalling pathways of SP binding to the NK1 receptor. Cell proliferation, inhibition of apoptosis and migration after activation of NK1R by SP (Adapted with permission from Ref. [58]. 2012 Rosso, M. et al. Previously modified from Ref. [64]. 2010 Munoz, M. et al.).

When SP is released, it exerts changes in peripheral tissues, resulting in mast-cell degranulation, vessel dilatation and increased vascular permeability, most of which are pathophysiological changes also found in acute inflammation [66]. In fact, NK1R antagonists have been proposed as a pain therapy [67] and as anti-inflammatory [68] drugs. We have reported that SP may be an important factor in the promotion and progression of different tumours [12]. SP, included in the tachykinin family of neuropeptides, binds to the NK1R receptor, regulating the intensity of noxious signals [69] and responsible for neurogenic inflammation [70]. As SP/NK1R binding activates different elements of the mitogen-activated protein kinase (MAPK) cascade (Figure 1) [71], our group have proposed the induction of mitogenesis as a relevant SP function. In fact, SP promotes cellular proliferation by the transactivation of the receptor tyrosine kinase epidermal growth factor receptor (EGFR) (Figure 1). We have demonstrated this event in several neoplastic cell lines, confirming that SP acts through NK1R as a mitogen. Contrarily, NK1R antagonists induced the apoptosis of tumour cells [66].

In a previous work, we reported both SP and NK1R expression in more than 90 out of 97 specimens of laryngeal epithelium close to laryngeal carcinoma [22]. We recently described that both SP and NK1R immunoreactivity, in oral epithelial cell cytoplasm, were significantly associated with the expression of Ki-67 in dysplastic epithelium [21]. Brener et al. [72] have also demonstrated SP and NK1R immunostaining in oral squamous cell carcinoma, describing a widespread expression of both proteins in the infiltrating lymphocytes and blood vessels, and also in the cell membrane, cytoplasm and nuclei of tumour cells. Our group have also reported that SP is widely expressed in laryngeal tumours and may be involved in malignant changes from epithelial dysplasia to carcinoma. Interestingly, we also found lymphocytes in normal laryngeal mucosa staining for SP whereas those present in lymph node metastases did not [22]. It is quite tempting to suggest SP secretion from those lymphocytes, linking chronic mucosal inflammation and cancer, as has already been described in neoplastic lymphocytes [73].

In addition, the SP/NK1R system could regulate the immune system and the immune response [74,75]. A number of different effective pathways, mediated by SP between the immune and the nervous system, have been described: from the central nervous system to the bloodstream, from sensory nerves, from autocrine/paracrine release, among others. Tachykinins also enhance the response of different inflammatory cells, including lymphocytes, monocytes and macrophages, mast cells or granulocytes, and lower concentrations of SP induce mucosal oedema, which can be reversed by NK1R antagonists [76]. Human dental pulps have been used extensively as models to study the precedent phenomena. Dental pulp seems to respond to occlusal trauma and to masticatory functions through neurogenic inflammatory processes in which SP plays a central role in the direct and indirect mechanisms of angiogenesis by triggering the NK1R receptors of fibroblasts, endothelial and inflammatory cells, leading to the formation of new blood vessels [77]. These actions are also potential oncogenic mechanisms, by which SP/NK1R could hypothetically regulate neoangiogenesis in head and neck cancer cells.

**Figure 2:**
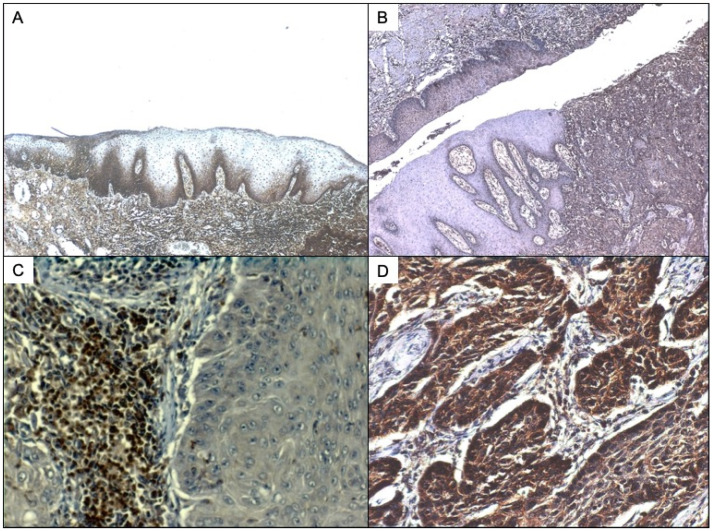
(**A**) SP immunostaining of oral non neoplastic epithelium. SP can be detected in this case both in the membrane of basal keratinocytes and also in the nuclei of subepithelial lymphocytes. The stroma also presents positive inflammatory infiltrate. (**B**) NK1R immunostaining of laryngeal carcinoma and dysplastic epithelium. Strong immunostaining for NK1R can also be detected in the lymphocytes under the basal lawyer of mucosa and tumour infiltrating lymphocytes. (**C**). SP immunostaining of laryngeal carcinoma and dysplastic epithelium with greater increase to show better the infiltrating lymphocytes of dysplastic epithelia. (**D**) SP immunostaining of laryngeal carcinoma showing, under greater zoom, the infiltrating nests expressing SP.

It has also been described that SP could originate from eosinophils and macrophages, as these cells up-regulate both SP and NK1R expression during acute and chronic inflammation [78,79]. As we have seen in laryngeal and oral non-neoplastic epithelium (Figure 2A), there is a strong SP expression in the keratinocytes of the basal layers, also present in the band-like lymphocytic inflammatory infiltration of the basal layer of the epithelium. When studying NK1R expression (Figure 2B), both dysplastic epithelium and invasive tumour showed immunostaining for the receptor, in which it was quite evident that NK1R is expressed in the tumour but not the adjacent non-dysplastic epithelium.

The proliferation of keratinocytes has been related to MAPK activation in various in-vitro studies [80]. It has also been reported that activation of the MAPK cascade may promote a hyperproliferative status of stem cells without terminal differentiation [81], which is characteristic of precancerous epithelium [82]. As SP can induce cell proliferation by transactivation of the receptor tyrosine kinase EGFR [12], it may promote oncogenesis by stimulating the cell proliferation of head and neck mucosal epithelium. We have detected SP and NK1R immunostaining in the cell membrane, and also SP in the cytoplasm and nuclei of epithelial cells in non-tumour epithelia adjacent to invasive carcinomas (Figure 2A,B) in both oral and laryngeal precancerous epithelium.

**Figure 3:**
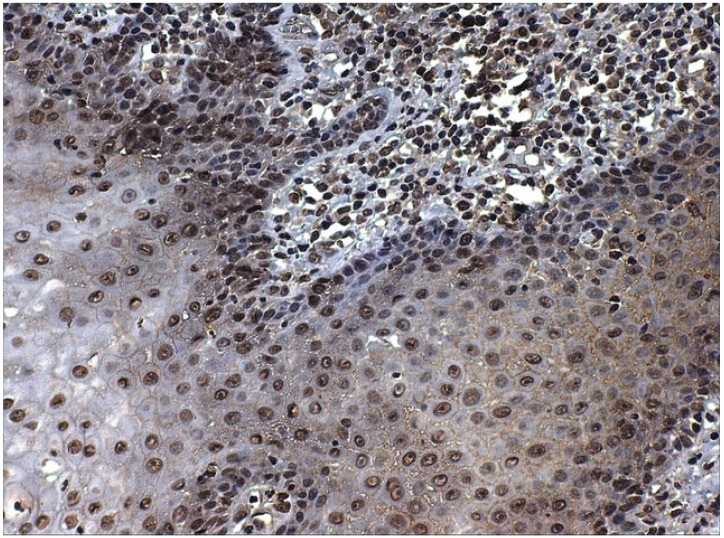
SP immunostaining of nuclei of epithelial cells in non-neoplastic epithelium. SP can be detected, in this case, both in keratinocytes and also in the nuclei of subepithelial lymphocytes.

**Figure 4:**
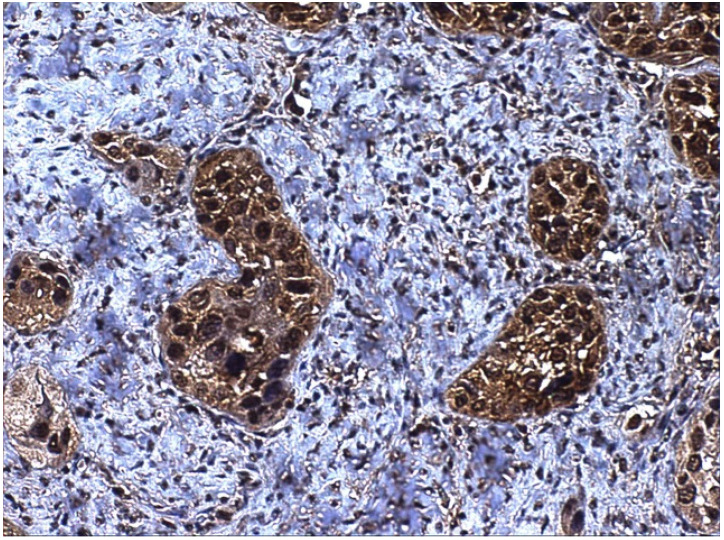
SP immunostaining of both cytoplasm and nuclei of neoplastic squamous cells in laryngeal carcinoma.

To our knowledge, no nuclear receptors for SP have been described, and we detected the nuclear expression of SP (Figure 3 and Figure 4), which may indicate the presence of nuclear receptors, as reported for other proteins (e.g., nuclear and membrane receptors have been described for melatonin) [83]. These novel receptors may activate the proliferation of the cells in an alternative way, and, in this context, SP could be considered a genetic neuromodulator [84]. SP regulates transcription factors, such as NF-kB, cytokines and chemokines, which are involved in inflammation [85], ERK1/2 in extracellular signal-regulated kinases, c-fos, c-jun and AP-1 in mitogenesis and cellular differentiation and hypoxia-inducible factor (HIF-1a) in angiogenesis (see for review Ortiz-Prieto et al. [86]). These interactions with NF-kB, ERK 1/2 and HIF1-alpha could potentially explain an association between SP and epithelial mesenchymal-transition, a phenomenon in which epithelial cells shed their epithelioid morphology to acquire a fibroblastoid phenotype, with increased cell migration and invasiveness [87]. Singh et al. [85] have very recently found that SP induced a strong up-regulation of the EMT-associated genes TWIST1, TWIST2, Gli, Patched and SHH, thereby activating the EMT phenomenon in different head and neck cancer cell lines. On the other hand, the relationship with HIF-1a could also potentially explain an association between SP and tumour hypoxia, a situation where tumour cells have been deprived of oxygen, acquiring hypoxic microenvironment changes, associated with extracellular matrix remodelling, increased cell migration, metastasis and multidrug resistance [88]. Although this interaction has not yet been investigated in head and neck cancer, Walczak-Drzewiecka et al. [89] found that, under hypoxic conditions, exposure of mast cells to SP resulted in a significant up-regulation of HIF1A expression, the key regulator of the cellular response to hypoxia. Future studies are needed to corroborate this hypothesis in head and neck cancer. SP, located in the tumour cell nucleus (Figure 4), may directly modify gene transcription or indirectly regulate the transcription factors of tumour epithelial cells. Lieb et al. [90], in this sense, have demonstrated that SP activated the transcription of NF-kB in human astrocytoma cells in a specific way. This activity required the mobilization of intracellular calcium and the formation of reactive oxygen intermediates as secondary messengers [91]. The possible regulation of other transcription factors involved in cancer (i.e., ERK1/2, c-myc, c-fos, c-jun, AP-1 and HIF-1α) and mediated by SP has also be hypothesized [66].

We have also reported SP expression in dysplasia/carcinoma in situ of both the oral cavity and larynx, proving that SP could be involved in the early stages thereof, prior to malignant transformation. We detected a strong relationship between SP expression in adjacent non-tumour epithelium and in oral carcinomas [72], thus, SP-mediated stimuli may be important for reaching the malignant phenotype. Our group has also proposed SP to be produced by lymphocytes in the epithelial cells [20]. Thus, lymphocytes playing an immunosurveillance role in the head and neck epithelia could represent a source of SP that, in some cases, may be responsible for malignant transformation and head and neck cancer progression. This mechanism has also been suggested by others for the malignant transformation of oral lichen planus, for instance [47]. In conclusion, SP is present in head and neck epithelia as hyperplastic or dysplastic, not only at the membrane or cytoplasm but also at the nucleus level. Further investigation will be necessary to investigate SP pathogenic pathways.

## 3. SP/NK1R in Head and Neck Cancer

### 3.1. Oral Potentially Malignant Disorders and Oral Cancer

SP/NK1R has been scarcely investigated in oral potentially malignant disorders [31]. Only one study has reported data on the expression of these biomarkers in the context oral lichen planus [20], showing positive expression in 98% (49 out of 50) and 14% (7 out of 50) of cases, respectively, for SP and NK1R. High positive relative frequencies were also found in cases of adjacent non-tumour epithelium to oral cancer, both for SP and NK1R (66.27% (55 out of 83 cases) and 21.69% (18 out of 83 cases), respectively) [21]. These results could indicate that the overexpression of SP and/or NK1R are early events during the initial steps of oral carcinogenesis. Future studies on other potentially malignant oral disorders and pre-malignant tissues are needed to confirm this hypothesis.

Our group have demonstrated, for the first time, SP and NK1R immunostaining in oral squamous cell carcinoma (OSCC), and both proteins were detected in oral mucosa, blood vessels and infiltrating lymphocytes. The presence of SP was documented in the cell membrane, cytoplasm and nucleus of the neoplastic cells [72].

When studying tumour-infiltrating lymphocytes (TILs) it could be proposed that SP may be responsible for an increase in their immunosurveillance response. However, as an SP source derives from an autocrine or paracrine release by B- and T-lymphocytes [91], SP could also be used by tumour cells as a mechanism to increase their invasive potential and proliferative capabilities. We were able to detect significant SP expression among different tumour tissue levels, supporting the theory of a diffusion pathway for SP from lymphocytes [79,92]. Another interesting finding, also corroborated in our study on laryngeal carcinomas, was the presence of nuclear SP immunostaining. The finding of SP located inside the tumour cell nuclei strongly suggest that SP might act to regulate tumour gene expression. It has also been reported that SP modulates the emotional conduct under control of the limbic system [93], and we suggest that SP in the cell nucleus could be modulating the different tumour cells’ abilities to progress by activating/inhibiting gene expression.

On the contrary, it has been proposed that the cytoplasmic localization of NK1R is explained by internalization once it binds SP [94], as observed in other tumour types [94,95]. In our research, aiming to study SP/NK1R in OSCC [72], we described a significantly higher immunostaining of SP in both cell membrane and cytoplasm than in cancer cells’ nuclei, supporting the association found with a higher cell proliferation index by measuring ki67 expression in tumour tissues, as NK1R, once internalized, promotes cell proliferation (Figure 1). From our results we suggested an increase in the number of NK1R receptors, a finding that has been reported elsewhere [91]. In different tumour phenotypes, an increase in the number of NK1R receptors in comparison with normal cells has been reported [96,97], as tumour cells bearing higher number of receptors for SP would, therefore, increase their ability to multiplicate. However, in normal cells these proliferative stimuli would normally lead to apoptosis (see for review Esteban et al. [12]), consequently malignant tumours must neutralize these apoptotic signals through opportunistic mechanisms of cell death resistance. Further studies are needed to ascertain how tumour cells neutralize these apoptotic signals. However, NK1R antagonists have now been developed, such as L-773060 or Aprepitant, currently available for the blockage of these stimuli, which also need further investigation. Our group demonstrated, in a wide variety of tumour cell lines, that SP induced neoplastic growth and the NK1R antagonists exerted antitumour activity, inducing apoptosis (as an example, cells lines from laryngeal carcinoma, melanoma, glioma, retinoblastoma, neuroblastoma, among others) [10,62,63,98,99]. We have repeatedly demonstrated that the antitumour effect is dose-dependent [11,62]. Our findings support further research to ascertain the role of NK1R antagonists such as Aprepitant.

The widespread expression of SP and NK1R in tumour blood vessels, another finding from our tumour studies focused on OSCC, should also be highlighted. It is important to state that NK1R expression has been reported in the blood vessels of most tumour types investigated to date [18], and angiogenesis is a key ability of tumour progression and metastasis. In this context, NK1R stimulation by SP has been reported as an alternative mechanism for tumour cells to create new blood vessels in the context of the tumour microenviroment [13].

In summary, the widespread presence of SP/NK1R in oral carcinomas strongly suggests their important role in cancer progression, and we also would like to highlight the need for clinical trials to check the potential use of NK1R antagonists, such Aprepitant, for the management of patients with oral cancer.

### 3.2. Laryngeal Cancer

Our group pioneered the research on SP/NK1R in laryngeal cancer. We first described SP-NK1R system in the Hep-2 laryngeal carcinoma cell line and demonstrated that NK1R activation, mediated by SP, induced the growth of human laryngeal cancer cells [65]. We have also reported SP and NK1R expression in laryngeal cancer for the first time [22], as the primary level study conducted by Hennig and coworkers [98] did not study squamous cell carcinoma in their pioneering work on SP receptors in human primary neoplasms.

**Figure 5:**
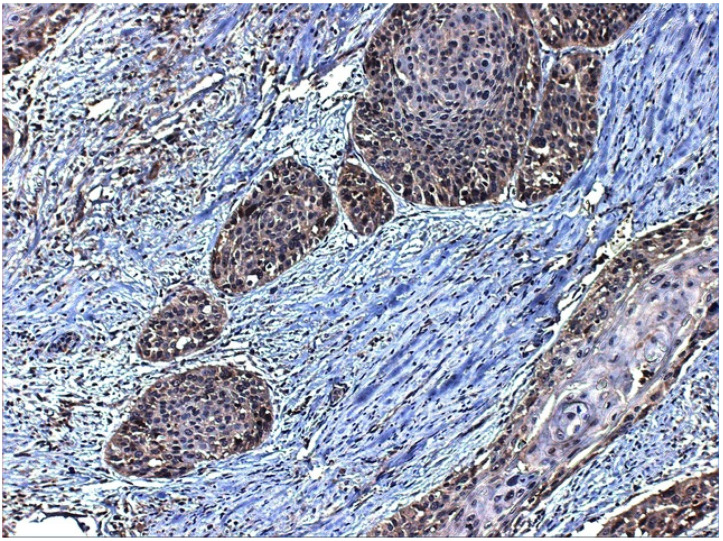
NK1R immunostaining of tumour cells in laryngeal squamous cell carcinoma. NK1R expression was detected mainly in the cytoplasm of the neoplastic cells. Interestingly, also fibroblasts and lymphocytes among the tumour nests displayed NK1R expression.

In our precedent work, 114 consecutive cases of patients who underwent surgery for laryngeal carcinoma were included, and we found SP immunostaining in the majority of the basal layers of non-neoplastic epithelium close to the tumour [22]. Additionally, most of the tumours and all the metastases displayed SP expression: 111 out of 114 (97.37%) primary tumours and all lymph node metastases studied were positive for SP (Figure 4). On the other side, 90 out of 97 (92.78%) samples were positive for NK1R (Figure 5). SP immunostaining was detected, both in the membrane and cytoplasm, which can be explained, as we have discussed before, on the basis of receptor internalization [94], but also it is important so state that SP was detected in the nuclei of tumour cells (Figure 4). SP is widely expressed in laryngeal tumours and may represent an important mechanism of tumour progression. In summary, SP could be the link between chronic mucosal inflammation and laryngeal cancer, as SP may be secreted by submucosal lymphocytes [73]. It is quite tempting to propose a role for SP in modulating the growth of laryngeal carcinomas and the treatment of laryngeal cancer with NK1R antagonists already used in clinical practice, such as Aprepitant.

### 3.3. Thyroid Cancer

Again, our group was recently the first to describe SP and NK1R in human normal thyroid tissues as has been done in thyroid cancer (TC) [23]. We studied a series of papillary, follicular, medullary and anaplastic tumours to include the most important and recognized varieties of thyroid cancer, as only medullary thyroid carcinomas had been reported before [98,100]. SP and NK1R were immunostained in all TC and non-tumour thyroid samples studied. Once again, as described for other tumours, SP/NK1R immunostaining was found to be higher in the tumours.

We documented the presence of SP/NK1R in the nucleus/cytoplasm of thyroid cancer cells [23]. Again, we hypothesize a role for SP as an epigenetic factor, regulating gene expression in these TC cells. On the other side, as SP was found in the cytoplasm of most thyroid cancer cells, it can be proposed a SP release in this tumour type [64,101].

As NK1R is detected in most of thyroid cancer cells, it could be suggested that SP overexpression could mediate and enhance mitogenic signalling pathways in this tumour type [18,102,103,104,105]. In this context, we have already discussed how the application of a knockdown gene-silencing method demonstrates that NK1R is essential for neoplastic cell viability [64]. SP may be released from TC cells and, to facilitate cell proliferation (autocrine mechanism through NK1R, as the fact that SP induces mitogenesis in human cancer cells) [58,101]. SP release from TC cells suggests a paracrine action on NK1R in endothelial cells, leading to proliferation, blood vessel growth and, thus, resulting in the rise of clinical neoplasms [58,106]. We can also suppose the SP release into the blood by an endocrine mechanism, increasing SP expression at the plasma level. All these suggestions, which are not difficult to test experimentally or in clinical practice, need further investigation.

### 3.4. Relationship with Environmental Factors

The quality of current evidence on the association between environmental factors, SP/NK1R and head and neck cancer is low, mainly due to the low number of published papers reporting observations in this regard. A recently published systematic review and meta-analysis has evidenced, for the first time, the differential expression of SP/NK1R across geographical areas of the world in patients with head and neck cancer [5]. This was an infrequent event in Asia, where 12.04% of patients overexpressed SP/NK1R, showing a significantly lower relative frequency in comparison with the other continents (65.03%; *p* = 0.007). Although confidence intervals were relatively wide for the Asian pooled proportions (95% confidence intervals = 0.00–35.87%), this event was obtained through the subgroup meta-analysis of a considerable number of primary-level studies (n = 8) [5], which increases the certainty and reliability of this result. The causes justifying this phenomenon are unknown; tobacco use could be a potential explanatory mechanism of this geographical difference. Currently, Asia is accepted to be the geographical region with the highest rate of tobacco consumption in the world [107]. Although the implications of tobacco consumption have not been researched in the context of the differential expression of SP/NK1R in head and neck cancer, an experimental study has reported the inhibition of NK1R-mediated signalling in response to second-hand tobacco smoke exposure in primate nucleus tractus solitarius neurons [108]. Further research is needed to corroborate whether involuntary environmental exposure to tobacco use, and the habit per se, may be the explanatory factor for the endemic differential of SP/NK1R.

Other important environmental risk factors playing an important role in head and neck cancer etiopathogenesis in some types of tumours, such as the Epstein–Barr virus [109], whose transmission could be linked to environmental diffusion, have not been analysed in depth in the context of the differential expression of SP/NK1R in head and neck cancer. Similarly, other environmental agents, such as pollution, have not been investigated so to date. Future studies are encouraged to investigate and increase the quality of evidence on the potential relationships between environmental factors and SP/NK1R, which could potentially have diagnostic, prognostic and/or therapeutic implications in these malignant neoplasms.

## 4. Conclusions

It is quite tempting to propose an important role for SP/NK1R system in the tumour progression of squamous cell carcinoma of the head and neck. The overexpression of the SP/NK1R system in epidermoid carcinoma of the head and neck open the door to the therapeutic intervention with the NK1R antagonist Aprepitant. The potential interaction of Aprepitant with other drugs (e.g., bosutinib, cabazitaxel, cyclophosphamide, dexamethasone, methylprednisolone, midazolam, oxycodone or tolbutamide, as demonstrated in a systematic review [110]) should also be considered during cancer treatment. Current evidence supports further research to ascertain the role of NK1R antagonists in head and neck cancer as a potential therapeutic strategy in future clinical trials.

## Data Availability

Data sharing is not applicable to this article as no new data were created or analyzed in this study.
